# Non-infected preterm parturition is related to increased concentrations of IL-6, IL-8 and MCP-1 in human cervix

**DOI:** 10.1186/1477-7827-3-39

**Published:** 2005-08-25

**Authors:** Susanne Abelin Törnblom, Aurelija Klimaviciute, Birgitta Byström, Milan Chromek, Annelie Brauner, Gunvor Ekman-Ordeberg

**Affiliations:** 1 Dept of Women and Child Health, Division for Obstetrics and Gynecology, Karolinska Institute, Karolinska University Hospital Solna, 171 76 Stockholm, Sweden; 2 Microbiology and Tumor Biology Center, Division for Clinical Microbiology, Karolinska Institute, Karolinska University Hospital Solna, 171 76 Stockholm, Sweden

## Abstract

**Background:**

Human cervical ripening is an inflammatory process. In labour at term the mRNA-levels and protein concentrations for interleukin-6 (IL-6) and IL-8 in cervix significantly increase. The aim of this study was to investigate if there are differences in the inflammatory process of preterm and term cervical ripening.

**Methods:**

Cervical biopsies from 50 singleton pregnant women without clinical signs of infection were allocated to four groups: preterm labour, term labour, preterm not in labour and term not in labour. The protein levels of IL-8, IL-6, monocyte chemotactic protein-1 (MCP-1), regulated upon activation normal t cells expressed and secreted (RANTES) and tumour necrosis factor-alpha (TNF-alpha) were quantified in tissue homogenates by ELISA or Immulite. The mRNA expression of IL-8, MCP-1 and RANTES was studied using RT-PCR. White blood cell count (WBC) and C-reactive protein (CRP) in the blood were determined. For determination of statistically significant differences between study groups Mann-Whitney U test or Kruskal-Wallis test were applied.

**Results:**

Protein concentrations of IL-8, IL-6, and MCP-1 were significantly increased during labour compared to non-labouring groups, whereas no changes were observed for RANTES and TNF-alpha. The mRNA levels of representative cytokines such as IL-8 and MCP-1 increased significantly during labour whereas RANTES mRNA expression remained unchanged. WBC and CRP were significantly higher in the labouring groups as compared to groups not in labour. For neither of the analysed cytokines, WBC or CRP levels were there any changes between preterm and term respective groups.

**Conclusion:**

Our findings indicate that non-infected preterm cervical ripening is an inflammatory process, just as cervical ripening at term, with cytokines as important mediators.

## Background

Preterm delivery occurs in approximately 6–15% of all pregnancies [1]. Furthermore, it accounts for 70–75% of neonatal mortality and morbidity [2]. The association between lower and upper genital tract infections and preterm delivery is well established [3]. However, in many cases preterm labour seems to be idiopathic [1]. The basic mechanisms underlying the initiation of both preterm and term cervical ripening and labour remain largely unknown. Both term and preterm labour requires cervical softening and regular myometrial contractions. Cervical softening is the result of an intensive remodelling process of the extracellular matrix (ECM). During cervical ripening the concentration of collagen decreases [4] and its physical state changes [5]. Additionally, the concentration of the main cervical proteoglycan decorin declines [6] in contrast to an increase of the mRNA expression and the protein concentration of the large proteoglycan versican [7]. Cytokines and several other mediators such as oestrogen, progesterone, nitric oxide and prostaglandins are involved in the human cervical ripening and the remodelling of extracellular matrix [8-11]. This remodelling process can therefore be regarded as an inflammatory reaction [12,13]. The density of immunoactive cells such as leucocytes and macrophages increases 6 to 10-fold at labour compared to first trimester pregnancy [14]. They are known producers of a variety of proinflammatory cytokines and matrix metalloproteinase's, and promote the cervical extracellular matrix degradation [13,15-17]. Furthermore, chemokines and cytokines are capable of attracting these immunoactive cells to the site of inflammation and are involved in human pregnancy and parturition [18,19].

Earlier studies have shown an increase of IL-8, IL-6 and TNF-α in human gestational membranes during spontaneous term labour [18,20,21]. Furthermore, the amniotic fluid levels of IL-8, RANTES, IL-6 and TNF-α [20] and MCP-1 [22] increase markedly at the onset of spontaneous labour at term. Cytokine levels in preterm parturition are widely analysed in relation to infection. However, it is estimated that less than 50% of the cases of preterm labour with elevated cytokines are due to an infection [23]. In placental cell cultures from non-infected preterm labour significantly larger amounts of IL-1β, IL-6, and TNF-α were registered compared to those shown by non-labouring women at term [24]. RANTES in non-infected amniotic fluid in preterm delivery is significantly higher compared to women with term delivery [25].

Only a few studies have investigated the role of the cytokines in human cervical tissue at term. The gene expression of IL-6 and IL-8 is significantly up regulated with a corresponding increase in the protein concentrations in patients in labour compared to not in labour [17,26,27]. Furthermore, animal studies have shown that IL-8 when applied intracervically induces cervical ripening in guinea pigs [28]. Studying the conditions at preterm parturition, Winkler et al. has shown that in the lower uterine segment, the IL-1β, IL-6 and IL-8 concentrations increased in relation to progressing cervical dilatation, whereas TNF-α remained unchanged [29]. Cytokines in the preterm cervix, to our knowledge, have not been investigated before.

We hypothesized that non-infected preterm cervical ripening and labour are associated with increased cytokine concentrations in the cervical tissue and increased inflammatory markers in the peripheral blood. The aim of this study was to investigate if there are any differences between inflammatory process during preterm and term cervical ripening. Therefore, protein concentrations of IL-6, IL-8, MCP-1, RANTES and TNF-α as well as mRNA expression of IL-8, MCP-1 and RANTES were determined in preterm and term groups. Furthermore, an analysis of inflammatory markers in peripheral circulation – White Blood Cell count (WBC) and C-reactive protein (CRP) – was undertaken.

## Methods

### Study Participants

A total of fifty women undergoing singleton pregnancies were included in the present investigation. The two study groups included 17 women in spontaneous preterm labour (PTL) and 8 not in labour at preterm (PTnotL). Premature delivery was defined as delivery before the 37^th ^week of gestation (Table 1). 14 women in term labour (TL) and 11 not in labour at term (TnotL) served as controls. Characterization of all study groups is summarized in Table 1. There were no significant differences regarding maternal age, parity and previous preterm births between the four groups.

**Table 1:** Characterization of the study groups

Parameter	Preterm labour (PTL)	Term labor (TL)	Preterm not in labour (PTnotL)	Term not in labor (TnotL)
N	17	14	8	11
Age (median, range)	28 (18–37)	28 (20–39)	32.5 (27–44)	29 (25–39)
Parity (median, range)	1 (0–3)	1 (0–3)	1 (0–7)	1 (0–2)
Previous preterm births	2	0	1	1
Gestational age in days (median, range)	242 (184–255)	281 (259–294)	219 (183–256)	270 (263–284)
Gestational age in full gestational weeks (range)	26+2 – 36+3	37+0 – 42+0	26+1 – 36+4	37+4 – 40+4
Treated with corticosteroids	2	0	5	0

The labour groups were in active labour and demonstrated a ripe cervix dilated >4 cm. In all patients delivered by caesarean section the assessment of cervical dilatation was established immediately before surgery, by the same obstetrician (SAT) through vaginal digital examination. The women in preterm labour were either delivered vaginally, or by emergency caesarean section due to malpresentation. The women in term labour were also either delivered vaginally or by emergency caesarean due to threatening foetal asphyxia. Women not in labour had unripe cervices (Bishop score <5p) and were delivered by caesarean section before the onset of labour. The preterm indications were suspected ablatio or intra-uterine growth retardation and the term indications were breech presentation, humanitarian or disproportion.

In all women clinical signs of infection were absent, during parturition as well as during the postpartal period.

### Specimen Collection

Immediately following parturition, a biopsy from the anterior cervical lip was taken transvaginally at the 12 o'clock position with scissors and tweezers. Our group has since 25 years applied this technique to get samples including squamous and cylindrical epithelium, vessels, glands and ECM. The samples were immediately frozen in liquid nitrogen and stored thereafter at -70°C until further investigation. A venous blood sample was taken for analysis of CRP and WBC.

Due to the limited amount of tissue from each woman, all analyses could not be performed on every sample.

The Local Ethics Committee of the Karolinska Institute approved the study (Ref. No. 97-089) and all women gave their informed consent.

### Tissue homogenisation

Frozen tissue was cut into small slices on a block of dry ice and transferred to a pre-chilled (liquid nitrogen) capsule containing Teflon coated tungsten ball. The capsule was kept in liquid nitrogen for two minutes and thereafter shaken in a dismembranation apparatus (Retsch KG, Haan, Germany) at full speed for two minutes. The procedure was repeated after intermediated freezing in liquid nitrogen until the tissue became powder. Hereafter followed either RNA extraction or measurement of cytokine concentrations.

### Measurement of cytokine concentrations

#### Tissue preparation

Following the tissue homogenisation, 1 ml of phosphate-buffered saline (PBS) was added. After centrifugation at 400 g for 5 min, supernatant was retrieved. Protein levels of IL-6, IL-8, TNF-α, MCP-1 and RANTES were expressed as picograms of cytokine per mg of total protein (pg/mg protein). Total protein concentration was determined by Bio-Rad's Protein Asssay, based on Bradford dye-binding procedure (Bio-Rad Laboratories Inc., Hercules, CA, USA), according to the manufacturer's instructions.

#### Determination of cytokine levels

Cytokine IL-6, IL-8 and TNF-α protein analysis were performed employing IMMULITE Automated Analyser (Diagnostic Products Corp., Los Angeles, CA, USA), using the commercially available immulite chemiluminescent enzyme immunometric assays (Immulite^®^, DPC, Los Angeles, CA, USA) according to the manufacturer's instructions. Analytical sensitivity and intra-assay and between assay coefficients of variation were respectively 2 pg/ml, 6.2% and 7.5% for IL-6; 2 pg/ml, 3.8% and 7.4% for IL-8; 1.7 pg/ml, 3.6% and 6.5% for TNF-α.

RANTES and MCP-1 concentrations in the supernatants of homogenized cervical samples were determined in duplicates using the quantitative sandwich enzyme-linked immunoassays (ELISA) by commercially available kits (Quantikine, R&D Systems, Minneapolis, MN, USA). The detailed procedures are described in the instruction booklets supplied by the manufacturers. The results were interpolated from the standard reference curve provided with each kit. The sensitivity of kits was 5 pg/ml for MCP-1 and 8 pg/ml for RANTES. The intra-assay and inter-assay coefficients of variation were respectively 7.8% and 6.7% for MCP-1; 3.6% and 10.3% for RANTES.

### Detection of mRNA by RT-PCR

RT-PCR was performed on the four groups of cervical biopsies; women in PTL (n = 17), TL (n = 14), PTnotL (n = 8) and TnotL (n = 10).

#### RNA extraction

Following the tissue homogenization (see above), total RNA was extracted with the help of Trizol reagent (Invitrogen, Carlsbad, CA, USA) according to the manufacturer's instructions. The RNA concentration was measured at 260/280 nm by the help of Eppendorf Bio Photometer (Eppendorf AG, Hamburg, Germany). The quality of total RNA was controlled by running on 1.5% agarose gels and visualised under ultraviolet light after ethidium bromide staining. Total RNA was subsequently stored at -70°C until further investigation.

### Reverse transcription (RT)

From each sample 1 μg RNA was taken, to which 1 μl (250 ng) of pd(N)_6 _Random Hexamer 5'-Phosphate primers (Amersham Biosciences, Pistacaway, NJ, USA), 1 μl of 10 mM dNTP (Amersham Biosciences) and sterile water was added to 12 μl. The mixture was incubated for 5 min at 65°C, cooled down and centrifuged. The reaction mixture consisting of 4 μl of 5 × First-Strand Buffer, 2 μl of 0.1 M DTT (Invitrogen, Carlsbad, California) and 1 μl (40 U/μl) Protector RNase Inhibitor (Roche, Mannheim, Germany) was added and incubated 2 min at 42°C. 1 μl (200 U/μl) of SuperScript™ Rnase H^- ^Reverse Transcriptase (Invitrogen, Carlsbad, California, USA) was added to each tube and mixed up. The RT step was carried out at 42°C for 50 min, followed by heating at 70°C for 15 min to inactivate the enzyme. The cDNA was stored at -70°C until used.

### RT-PCR

All gene-specific primers (Table 2) were obtained from Invitrogen (Carlsbad, California, USA). Ribosomic 28S was used as housekeeping gene. PCR was performed on 2 μl cDNA using Master Taq kit (Eppendorf, Hamburg, Germany) in a 25 μl reaction mixture containing 2.5 μl 10 × PCR buffer, 0.625 8 mM dNTP, 0.15 μl Taq polymerase (5 U/μl), 1 μl of specific primers (5 μM) and water. The PCR reaction was run in Eppendorf Mastercycler^® ^gradient (Eppendorf AG, Hamburg, Germany). Repeated experiments were performed to ensure that the PCR reaction was within the linear phase. 1 min at 95°C was followed by cycles of 1 min denaturation at 94°C, 1 min of annealing and 1 min of extension at 72°C. The final extension lasted for 5 min at 72°C and thereafter cooling to 4°C. The number of cycles and annealing temperatures for each primer are presented in table 2.

**Table 2:** Description of the primers used for RT-PCR. The sequences, the number of cycles, annealing temperature (Tm) and product size of primers used for RT-PCR analysis.

Genes	5' Primer	3' Primer	Cycles	Tm °C	Size bp
IL-8	TCTCTTGGCAGCCTTCCT	AATTCTCAGCCTCTTCAAAAACTT	34	61	276
MCP-1	CTCTGCCGCCCTTCTGTGCC	GTCTTCGGAGTTTGGGTTTGC	28	61	288
RANTES	CGGCACGCCTCGCTGTCATC	TGTACTCCCGAACCCATTT	34	61	240
28S	GTGCAGATCTTGGTGGTAGTAGC	AGAGCCAATCCTTATCCCGAAGTT	19	58	552

### Semi-quantification of IL-8, MCP-1 and RANTES mRNA

The PCR products were separated by electrophoresis on a 1.5% agarose gel (Amersham Biosciences AB, Uppsala, Sweden). Following staining with ethidium bromide, the gels were photographed and band intensity measured under UV light using Gel Doc 2000 (BioRad, Hercules, CA, USA). The specific mRNA level of every sample was expressed as the product's intensity, divided by the housekeeping gene 28S intensity (the product/28S intensity ratio).

The identity of PCR products was confirmed by sequencing them at KISeq, Center for Genomics and Bioinformatics, Karolinska Institutet.

### Determination of WBC and CRP

WBC and CRP were determined in blood in the routine laboratory of Karolinska University Hospital Solna (Stockholm, Sweden).

### Statistical Analysis

Comparison between two groups was performed using the Mann-Whitney U test. When all four groups were compared, the Kruskal-Wallis test was applied, followed by multiple comparison with Bonferroni correction. The level of significance was set at p < 0.05. Calculations were performed employing STATISTICA 6.0 software (StatSoft Inc, Tulsa, OK, USA).

## Results

### Protein concentrations of cytokines

Every analysed sample revealed detectable levels of proteins.

There were no significant differences between preterm and term respective groups, but differences reached significance when comparing groups in labour with not in labour.

**Figure 1:**
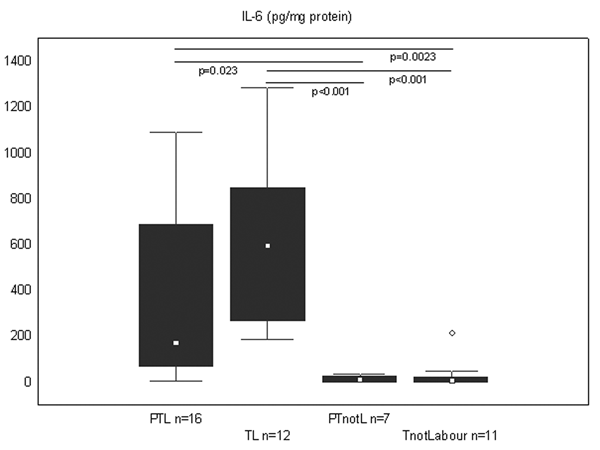
**IL-6 concentration in the cervical tissue in all study groups**. Protein concentration of IL-6 is expressed in picograms/mg of total protein. The groups are: preterm labour (PTL), term labour (TL), preterm not in labour (PTnotL), term not in labour (TnotL). The number of patients analysed in each group is marked in each bar in the bar chart. The box represents median value with 25%–75% of all data falling within the box. The whiskers extend to the non-outlier range. Outliers are marked as circles. Significant differences between the groups are shown above the box plots.

The concentration of IL-6 was significantly higher in the PTL versus the PTnotL (p = 0.02) and TnotL (p = 0.002). Similarly, concentration was significantly higher in the TL group compared to groups not in labour (Figure 1).

**Figure 2:**
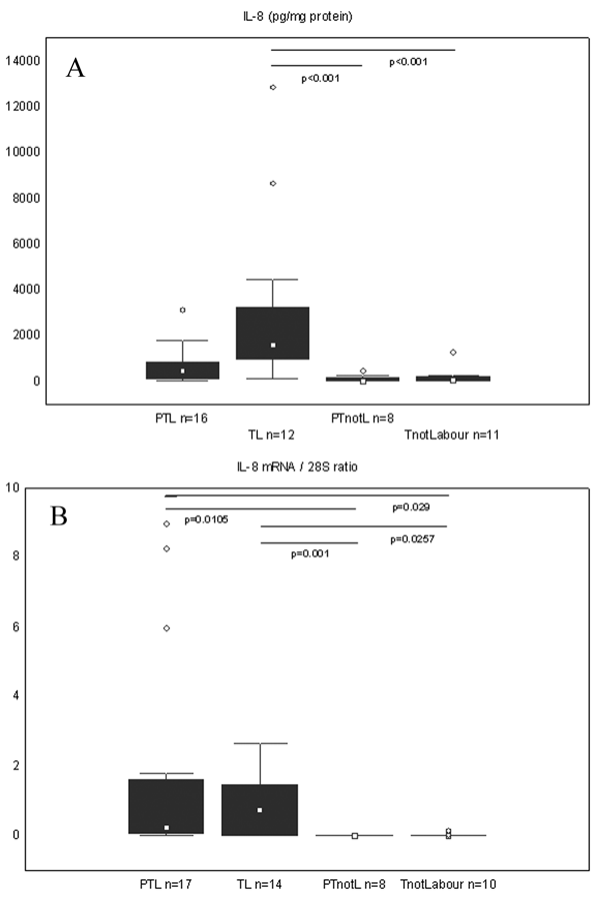
**Protein concentration and mRNA expression of IL-8 in the cervical tissue**. Box and whisker plots represent (A) the protein concentration of IL-8 (picograms/mg of total protein) and (B) expression of the IL-8 mRNA (expressed as the product/28S intensity ratio) in the cervical tissue of the study groups. The groups are: preterm labour (PTL), term labour (TL), preterm not in labour (PTnotL), term not in labour (TnotL). The number of patients analysed in each group is marked in each bar in the bar chart. The box represents median value with 25%–75% of all data falling within the box. The whiskers extend to the non-outlier range. Outliers are marked as circles. Significant differences between the groups are shown above the box plots.

Concentrations of IL-8 were significantly higher in the TL group compared to the PTnotL and the TnotL (p < 0.01). Although the same tendency was noted in the preterm groups, it didn't reach statistical significance (Figure 2A).

**Figure 3:**
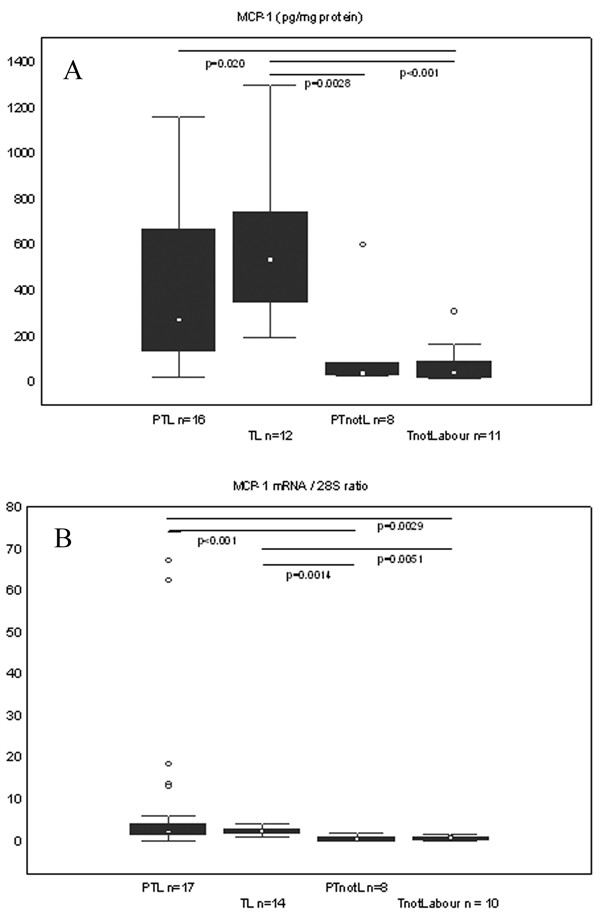
**Protein concentration and mRNA expression of MCP-1 in the cervical tissue**. Box and whisker plots represent (A) the protein concentration of MCP-1 (picograms/mg of total protein) and (B) expression of the MCP-1 mRNA (expressed as the product/28S intensity ratio) in the cervical tissue of the study groups. The groups are: preterm labour (PTL), term labour (TL), preterm not in labour (PTnotL), term not in labour (TnotL). The number of patients analysed in each group is marked in each bar in the bar chart. The box represents median value with 25%–75% of all data falling within the box. The whiskers extend to the non-outlier range. Outliers are marked as circles. Significant differences between the groups are shown above the box plots.

MCP-1 protein levels showed a tendency to be higher in the PTL compared to PTnotL, but did not reach statistical significance, whereas in the PTL was significantly higher compared to TnotL (p = 0.02). The concentration of MCP-1 was significantly higher in the TL group than in groups not in labour (Figure 3A).

Even more significant differences (p < 0.0001) between labouring and not labouring groups were achieved when it was looked upon the data irrespective gestational age (Table 3).

**Table 3:** In labour group compared with not in labour group. In labour group includes preterm labour (PTL) and term labour (TL) groups, not in labour group includes preterm not in labour (PTnotL) and term not in labour (TnotL) groups. Protein concentrations are expressed in pg/mg of total protein. Expression of mRNA is the product/28S intensity ratio. Significant differences between the groups were determined using Mann-Whitney U Test. NS-statistically not significant difference.

Measured factor	In Labour Group (PTL and TL)				Not in Labour Group (PTnotL and TnotL)				p value
		
	Median	Min	Max	N	Median	Min	Max	N	
IL-6 protein	396.7	7.7	1286.4	28	5.9	2.1	215.0	18	<0.0001
IL-8 protein	818.9	13.9	1288.1	28	71.0	4.8	1278.9	19	<0.0001
MCP-1 protein	420.8	21.2	1293.5	28	39.5	15.4	598.7	19	<0.0001
TNF-α protein	4.6	0.8	31.4	18	3.5	1.7	7.8	10	NS
RANTES protein	576.5	165.25	1075.3	18	439.5	141.0	869.6	10	NS
IL-8 mRNA	0.4	0	8.9	31	0	0	0.1	18	<0.0001
MCP-1 mRNA	1.9	0	67.2	31	0.4	0	1.4	18	<0.0001
RANTES mRNA	1.3	0	54.0	31	1.1	0.03	13.8	18	NS
WBC	15.9	7.3	28.4	21	9.6	6.9	14.1	14	<0.001
CRP	13.0	6.9	123.0	18	7.5	6.9	17.0	8	0.0221

Interestingly, there were no significant changes between preterm or term groups as regards the protein concentrations of TNF-α or RANTES (data not shown, combined data – table 3).

### IL-8, MCP-1 and RANTES mRNA expression

In line with protein data, there were no differences registered in the representative cytokine mRNA expression between preterm and term respective groups. On the other hand, mRNA expression of IL-8 and MCP-1 was significantly higher in labour compared to not in labour groups.

IL-8 mRNA levels were significantly higher in the PTL group compared to the PTnotL (p = 0.01) and to the TnotL group (p = 0.03). Furthermore, there were significant differences comparing TL with groups not in labour (Figure 2B).

In line with the IL-8 expression, MCP-1 mRNA levels were significantly higher in labouring groups compared to not labouring groups (Figure 3B).

In line with protein data, differences were even more significant (p < 0.0001) comparing labouring (including PTL and TL) and non-labouring groups (including PTnotL and TnotL) (Table 3).

Similarly to the protein levels, no significant changes were found in RANTES mRNA expression (data not shown, combined data – table 3).

### WBC and CRP levels

In line with cytokine levels, there were no differences in WBC (10^9^/l) and CRP (mg/l) levels between preterm and term respective groups. However, higher levels of inflammatory markers were found in the labouring groups compared to the nonlabouring groups. WBC was significantly (p = 0.017) higher in the preterm labour group with a median value of 15.9 (range 9.2–24.3) compared to the preterm group not in labour, where the median value was 8.6 (6.9–13.6). The same tendency was seen in the term groups, where median and range in the TL group was 15.65 (7.3–28.4) and in the not labour group 10.0 (7.4–14.0) respectively, however without statistical significance.

CRP levels also revealed the same tendency, although differences didn't reach statistical significance. Median and range in the groups were respectively 13.0 (6.9–32.0) in PTL, 13.0 (6.9–123.0) in TL, 12.5 (8.0–17.0) in PTnotL and 7.0 (6.9–10.0) in TnotL.

**Figure 4:**
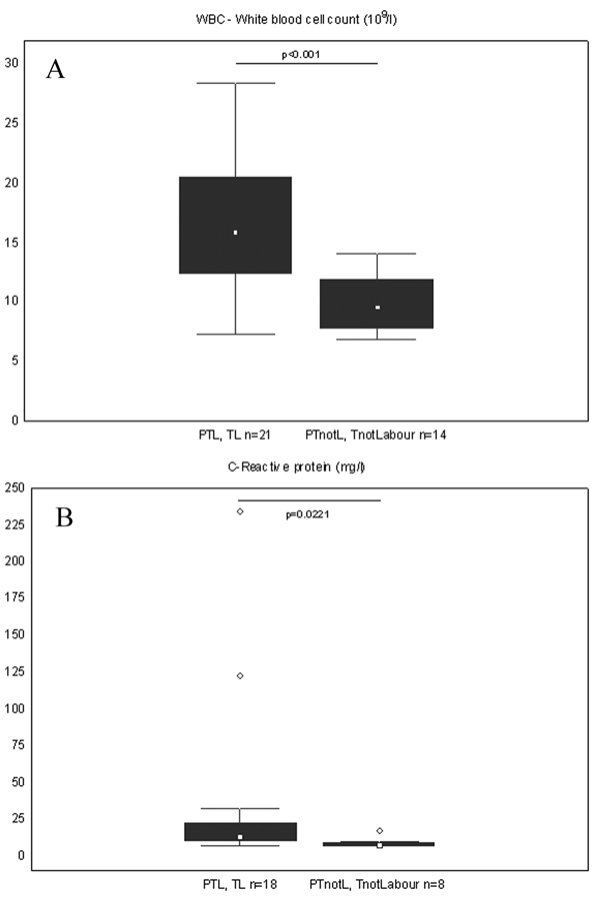
**WBC and CRP levels in labour compared to not in labour group**. Box and whisker plots represent (A) white blood cell count (10^9^/l) (B) C-reactive protein (mg/l) in the blood of women in the study groups. The two groups are: In labour (including preterm labour (PTL) and term labour (TL) and not in labour (including preterm not in labour (PTnotL) and term not in labour (TnotLabour). The number of patients analysed in each group is marked in each bar in the bar chart. The box represents median value with 25%–75% of all data falling within the box. The whiskers extend to the non-outlier range. Outliers are marked as circles. Significant differences between the groups are shown above the box plots.

WBC and CRP were significantly higher when compared the labouring groups (PTL and TL) to the groups not in labour (PTnotL and TnotL) (p < 0.001 and p = 0.02 respectively) irrespective to gestational age (Figure 4, Table 3).

## Discussion

To our knowledge, this is the first investigation on cytokines in preterm cervical tissues in non-infected subjects.

The hypothesis that clinically non-infectious preterm cervical ripening and labour is associated with increased cytokine levels was tested and verified for IL-8, IL-6 and MCP-1 protein concentrations. This was not valid for RANTES or TNF-α protein concentrations which all remained unchanged.

Interestingly, no significant differences were revealed between the preterm and term labour samples (ripe cervices) or between preterm and term not in labour (unripe cervices) samples. In other words, this study could not identify any differences related to the gestational age, while differences were found related to the cervical state.

Our findings concerning term cervical ripening agree with those in previous studies, where increase in IL-8 and IL-6 concentrations and mRNA expression was found in cervical tissue in labouring compared to not labouring subjects [17,26,27,30]. Analogous changes were registered in the lower uterine segment [31-33].

Looking at the changes in preterm labour, Winkler et al has shown similarities in IL-1β, IL-6 and IL-8 increase in the lower uterine segment during preterm and term parturition [29]. The only observed difference was that this increase at preterm starts at earlier stages of cervical dilatation [29,34]. Even though the lower uterine segment contains lower amounts of extracellular matrix, undergoes less intense changes and has another function than the cervix during pregnancy and parturition, the remodelling process there may still somewhat reflect events in the cervix [35,36]. Our findings in the preterm cervical tissue agree with the study on the lower uterine segment, but we are not able to judge at which stage of cervical dilatation the increase in cytokine levels begins.

In our earlier study, we have shown a decrease in 15-hydroxyprostaglandin dehydrogenase expression related to the cervical state irrespective of gestational age [37]. All these findings suggest that cervical ripening at preterm is a similar process as at term. This similarity can be further confirmed, looking upon our results allocated into two groups: in labour (containing preterm and term labour) and not in labour (containing preterm and term not in labour), where even more significant differences between labouring and not labouring groups are achieved. Analogous results are seen in WBC count and CRP levels, where these markers seem to rise significantly in the labouring groups compared to the groups not in labour. This suggests that local inflammatory process in the cervix can be reflected in the peripheral blood. IL-6 is a prominent stimulator of the acute phase response in inflammatory reactions and stimulates CRP. Taking that into account, it is logical that when we identify a significant rise in IL-6 in the labour groups we can expect a concomitant rise in the CRP level. The WBC count and the CRP level are used routinely as markers of infection in daily clinical use, but our findings may suggest that WBC and CRP could probably be markers of active labour without any infection. Further studies with larger number of patients are needed to confirm this.

All these findings cannot answer the question which signals cause the cytokine increase in the labouring cervix. Preterm labour in correlation with infection is extensively studied. A significant cytokine increase is seen in gestational membranes, amniotic and cervicovaginal fluid in infected preterm labour [3,21,38-40]. In our study, none of the women showed clinical or laboratory signs of infection before or after labour. This could also account for the unchanged levels of TNF-α in our and Winkler et al study [29], as TNF-α is shown to be related to infection in the amniotic fluid [41]. However, no correlation was found between chorioamnionitis and elevated IL-6 and IL-8 levels of amniotic fluid in term pregnant women [42]. Our findings are in line with earlier studies, where cytokine increase was seen in non infected preterm parturition [23,24,29] suggest that some other signals than infection could be responsible for starting preterm cervical ripening and labour process. The down-regulation of progesterone receptors and oestrogen receptor α with significant up-regulation of oestrogen receptor β (ERβ) at term pregnant cervix [8,43] may be involved. Furthermore, there is a possibility of direct effect of estrogens via ERβ on cervical leukocytes [44], which are a major source of proinflammatory cytokines in the cervix during labour [45]. Foetal fibronectine could be also involved in foetal-maternal signalling, as its level in the cervicovaginal fluid rises in line with the ripening of cervix and is elevated at preterm birth [46-48]. It can also be localised in the cervical epithelium as well as IL-8 [26,47]. Corticotropine releasing hormone (CRH) is elevated in maternal serum in preterm labour [49] and there is possible cross-regulation between CRH and cytokines, but earlier studies show controversial results [50-52]. Further investigations are required to clarify the involvement of these factors in the preterm cervical ripening and labour and their relation with the cytokine increase.

## Conclusion

In conclusion, preterm cervical ripening can be likened to an inflammatory process with cytokines as important mediators, corresponding to the process at term cervical ripening. The local changes in the cervix may be reflected in the peripheral blood as an increase in WBC count and CRP.

## Authors' contributions

SAT have selected and recruited the patients, collected all the biopsies, participated in the design of the study, did a part of laboratory analyses, drafting of the manuscript. AK participated in the analysis and discussion of the results, performed a part of statistical analysis and drafted the manuscript. BB participated in design of the study, did RT-PCR, ELISA, Immulite analyses, participated in the discussion of results and revising the manuscript. MC participated in the design of the study, RT-PCR analysis, discussion of the results and statistical analysis. AB participated in the design of the study, discussion of the results, revising the manuscript. GEO participated in the design of the study, analysis and discussion of the results, drafting and critical revising of the manuscript. All authors read and approved the final manuscript.
